# Impact of Thin-Ideals in Influencer Posts Promoting Healthy vs. Unhealthy Foods on Tweens’ Healthy Food Choice Behavior

**DOI:** 10.3389/fpsyg.2022.789069

**Published:** 2022-04-04

**Authors:** Steffi De Jans, Liselot Hudders, Brigitte Naderer, Valentina De Pauw

**Affiliations:** ^1^Department of Communication Sciences, Ghent University, Ghent, Belgium; ^2^Department of Media and Communication, Ludwig-Maximilians-Universität (LMU) München, Munich, Germany

**Keywords:** Influencer marketing, food marketing, healthy eating behavior, tweens, thin-ideals

## Abstract

The current study examines how social media influencers can be deployed to promote healthy food choice behavior among tweens. In particular, we investigated whether tweens’ healthy food choice behavior can be stimulated by using a thin-ideal influencer in a sponsored influencer post promoting unhealthy vs. healthy food. A two-by-two, between-subjects experimental study (influencer weight: thin-ideal vs. overweight; snack-type: unhealthy vs. healthy) was conducted with 146 tweens (11–13 years old, 73 boys). Results show that tweens’ choice for a healthy snack was higher when a (female) overweight influencer promoted an unhealthy snack (compared to a healthy snack). Using a thin-ideal influencer promoting an unhealthy vs. healthy snack did not affect tweens’ healthy food choices. While there were no interaction effects of influencer weight and snack type on source effects (influencer credibility, influencer admiration, and *trans*-parasocial interactions), the results did show that the influencer was perceived as less credible and was admired less when she was overweight vs. when she had a thin-ideal body-type.

## Introduction

As food preferences start to develop early in life, food marketers heavily target children, tweens, and teens (Tan et al., 2018), whereby they are exposed to a great number of food advertisements on different media. To break through the advertising clutter, new tactics emerge to attract children’s and adolescents’ attention and impact on their food preferences and choices. Especially the popularity of social media gave rise to new platforms and formats to endorse food products to the young audience. One of those new advertising formats is influencer marketing, which refers to social media influencers promoting brands or products on their social media profiles in return for compensation (Hudders et al., 2021). Influencer marketing has been shown to heavily target children (De Veirman et al., 2019). Recent content analyses show that much of the foods that are endorsed on social media are non-core foods or unhealthy foods (Tan et al., 2018; Coates et al., 2019a). Using a diary study where adolescents had to capture their exposure to food marketing messages on social media, Qutteina et al. (2019) showcased that adolescents were mainly exposed to non-core foods in oversized portions on social media.

This raises concerns as young audiences are less successful in resisting food marketing temptations compared to adults, as their knowledge and skills to critically reflect on those marketing attempts, referred to as persuasion knowledge (Friestad and Wright, 1994), are still developing. They also often lack the motivation to resist such attempts (Harris et al., 2009; Hudders et al., 2017). Moreover, children’s liking of food products is also largely determined by their familiarity with food (Cooke, 2007). Accordingly, food marketing, mainly showing unhealthy food products, is one of the factors that might contribute to childhood obesity (Folkvord et al., 2016), which is considered as one of the most serious global public health challenges of the 21st century. The World Health Organization (WHO) states that 39 million children under the age of 5 years were overweight or obese in 2020 and over 340 million minors between 5 and 19 years were overweight or obese in 2016 (WHO, 2020). Childhood obesity poses both physical and psychological health risks, that can occur both immediately and in the future (Sahoo and Garg, 2012). Thus, children need to adopt a healthy diet. Children’s and adolescents’ perceived benefits of healthy eating are improvement of cognitive and physical performance, fitness, endurance, psychological benefits, feeling good physically, and the production of energy (O’dea, 2003).

Recently, children and adolescents are also increasingly exposed to healthy foods and behaviors on social media, such as influencers promoting veganism (Schmuck, 2021), fitspiration (i.e., images and text promoting health and fitness), and thinspiration (i.e., images and text promoting thinness) (Alberga et al., 2018). However, extant research has mainly examined how influencer marketing promoting unhealthy foods affects children’s and adolescents’ food choice behavior and has shown that influencer marketing is effective in promoting unhealthy foods and increasing caloric intake among children (Coates et al., 2019b; Smit et al., 2019). Although children and adolescents are also increasingly exposed to healthy content on social media, there is hardly any research on how influencer marketing can be used to promote healthy foods to this target group (Folkvord and Hermans, 2020; De Jans et al., 2021). Limited prior studies revealed that mere exposure to influencer marketing for healthy foods does not increase children’s and adolescents’ choice of healthy foods (Coates et al., 2019a; Folkvord and de Bruijne, 2020). Recently, De Jans et al. (2021) did show that an influencer’s lifestyle (sedentary vs. athletic) affects children’s choice for healthy food, indicating that influencers’ characteristics can have an impact on how receptive children are to promoted food options.

Therefore, by examining the impact of the weight of the social media influencer (thin-ideal vs. overweight), the current study investigated another personal characteristic of an influencer promoting healthy vs. unhealthy foods on tweens’ (i.e., young people between 10 and 13 years of age; Enochsson, 2007; McArthur et al., 2021) choice for healthy foods. Thus, this study aims to examine how social media influencers can be used to endorse healthy (vs. unhealthy) foods to tweens. In particular, the current study investigates whether a social media influencer’s physical characteristics (i.e., influencer weight) can affect tweens’ food choice behavior. How an influencer’s weight and the promoted snack-type (healthy vs. unhealthy) affect the evaluation of the influencer (i.e., source effects) is also examined.

## Theoretical Framework

### How Children and Adolescents Are Persuaded by Influencer Marketing for Unhealthy Foods

Previous research has already examined how influencer marketing promoting unhealthy foods can have an impact on children’s and adolescents’ food choices. Although children and adolescents themselves believe that they can resist influencer marketing promoting unhealthy foods (Coates et al., 2020), research univocally shows that influencer marketing for unhealthy foods affects their unhealthy food preferences, choices, and intake. For example, Coates et al. (2019b) showed that exposure to influencer marketing on YouTube promoting an unhealthy snack (with and without an advertising disclosure, i.e., a label signaling the presence of advertising) resulted in children (9–11 years) consuming more of the advertised snack compared to the alternative snack, while there was no difference between the consumption of the advertised and alternative snacks in the control condition (no food marketing). Using longitudinal survey data, Smit et al. (2019) found that the frequency of watching vlogs of social media influencers increases the consumption of unhealthy beverages (but not the consumption of unhealthy snacks) among children and tweens between 8 and 12 years. In addition, a study by Coates et al. (2019a), examining influencer marketing on Instagram, showed that children’s (9–11 years) overall caloric intake and their intake of unhealthy snacks increased after viewing influencer marketing promoting unhealthy snacks compared to the control condition where non-food products were promoted.

The persuasive impact of food marketing for unhealthy foods can be explained by the Reactivity to Embedded Food Cues in Advertising Model (REFCAM) (Folkvord et al., 2016). The REFCAM puts forward a two-step process of food advertising. First, physiological and psychological reactions (such as the activation of a central appetitive state) to unhealthy foods are induced through food cues in food advertising (a process referred to as the advertising effect process). These reactions subsequently lead to a reciprocal relationship with unhealthy eating behavior (which is labeled as the incentive-sensitization process) (Folkvord et al., 2016).

In addition, the model proposes that children’s and adolescents’ susceptibility to food advertising is not only determined by their dispositional factors (such as weight and impulsivity), but that also message factors (such as the media context in which the advertising is integrated) moderate the effect of food advertising for unhealthy foods (Folkvord et al., 2016). Influencer marketing is an embedded advertising format, as these sponsored posts have the same layout and design as the other entertaining, non-sponsored posts of the social media influencers (De Jans and Hudders, 2020). This makes it difficult for viewers of these posts to recognize the sponsored posts as advertising and activate critical reflections toward the food advertising, which increases the food advertising’s effectiveness (Folkvord et al., 2016). This is especially challenging for children and adolescents, as their persuasion knowledge is not yet matured, making them even more susceptible to food advertising for unhealthy foods (Hudders et al., 2021).

### Using Social Media Influencers to Promote Healthy Food Choice Behavior

Due to the findings that influencer marketing is effective in promoting unhealthy foods, researchers started investigating whether this marketing tactic can also be used to promote healthy nutrition. These results are, however, not so univocal. Prior research shows that merely exposing children and adolescents to influencer marketing promoting healthy foods does not affect their healthy food choice behavior. For instance, besides showing that influencer marketing on Instagram promoting unhealthy foods increases children’s intake of unhealthy snacks, the study of Coates et al. (2019a) also showed that influencer marketing promoting healthy foods did not affect children’s (9–11 years) food intake. In line with this, Folkvord and de Bruijne (2020) found that the promotion of vegetables in influencer posts on Instagram did not affect adolescents’ (13–16 years) intake of vegetables (compared to a condition where an unhealthy snack was promoted and a control condition where no food product was promoted).

In line with the REFCAM (Folkvord et al., 2016), Folkvord (2019) proposed the Healthy Food Promotion Model explaining the effectiveness of advertising promoting healthy food behavior. First, the model suggests that through the promotion of healthy food consumers’ attention toward the value of healthy foods will be increased, which will lead to a reciprocal relationship with dietary intake. This will subsequently lead to an enhanced intake of healthy foods and therefore a habit formation concerning the intake of healthy foods. Finally, this habit of eating healthy foods for longer periods will eventually result in improved health states (Folkvord, 2019).

However, as extant research shows that the mere exposure to influencer marketing for healthy foods does not directly affect children’s and adolescents’ healthy food choice behavior, researchers very recently started investigating the factors that influencer marketing promoting healthy foods can have an impact on children’s and adolescents’ healthy food choice behavior. This is based on the Healthy Food Promotion Model (Folkvord, 2019), which suggests that potential individual and contextual factors might affect the relationship between healthy food promotion and dietary behavior. For example, the studies of De Jans et al. (2021) and Folkvord et al. (2021) investigated contextual factors related to influencers. More specifically, they examined whether a social media influencer promoting a fit lifestyle can affect children’s healthy food choice behavior. Folkvord et al. (2021) showed that using a popular, fit influencer led to a higher healthy food brand attitude and purchase intention among adults than a fictitious fit influencer *via* parasocial interaction. In addition, De Jans et al. (2021) found that using a social media influencer with a sedentary lifestyle (compared to an influencer with an athletic lifestyle) promoting food products resulted in more children and tweens (8–12 years) opting for healthy food option. Children’s and tweens’ healthy food choice behavior was higher when a social media influencer with a sedentary lifestyle (compared to an athletic lifestyle) promoted an unhealthy food product. Thus, these studies indicate that the characteristics of the social media influencer can affect healthy food choice behavior among minors and adults.

The current study, therefore, examines another personal characteristic of the social media influencer as a contextual factor possibly affecting the relationship between healthy food promotion and healthy food behavior, namely, the weight of the influencer. In particular, this study examines and compares the impact of a thin-ideal influencer vs. an overweight influencer in promoting unhealthy vs. healthy foods on tweens’ healthy food choice behavior. Particularly on social media, conventional thin-ideals are increasingly challenged and there is a positive trend to more body diversity (Stewart and Ogden, 2021). At the same time, there is still a high stigmatization of overweight and obese people that is already deeply rooted in children’s assessments of others (Tiggemann and Anesbury, 2000). Thus, it is valuable to examine how the influencers’ body type affects their promotional efforts.

### Thin-Ideals in Influencer Marketing Promoting Healthy Foods

Previous advertising research has already examined how the size or weight of a model affects advertising effectiveness and health outcomes. Some studies have shown that using a thin model in advertising is not more effective in terms of advertising effects compared to other-sized models (Halliwell and Dittmar, 2004; Sohn and Youn, 2013; Roberts and Roberts, 2015), other studies do suggest that using an endorser with ideal body-size is most effective (Roozen, 2014). Nonetheless, research assumes that these results might also depend on the characteristics of the message’s receiver. For example, consumers who have internalized the thin-ideal are more receptive to thin models (Halliwell and Dittmar, 2004; Roberts and Roberts, 2015), and especially vulnerable youth may experience negative consequences from exposure to thin-ideal images (Stice et al., 2001). Besides its impact on advertising effectiveness, a recent study by Stewart and Ogden (2021) showcases how using diverse body types in media messages was able to decrease negative weight bias, yet using thin-ideals was most effective in increasing intentions to eat healthy in the adult, female sample. In addition, exposure to media displaying the thin-ideal body size is also linked to body dissatisfaction and body image disturbance, especially among girls and women (Hargreaves and Tiggemann, 2003; Grabe et al., 2008).

While there is the trend to more body diversity, thin-ideal images are still omnipresent on social media. A popular trend on social media among social media influencers is thinspiration. This refers to the sharing of thin-ideal images (through the presentation of images that contain thin bodies) on social media to inspire certain body ideals and encourage followers and other social media users to be thin (Talbot et al., 2017). Exposure to thinspiration on social media has been shown to reduce body satisfaction (e.g., Yee et al., 2020; Dignard and Jarry, 2021) and was also connected to eating disorder symptoms (Rodgers et al., 2016). Thus, past research has mainly examined the negative consequences associated with thin-ideal images on social media. This study aims to examine whether the usage of thin-ideal endorsers in an influencer context can also be deployed to bring about a positive impact (namely tweens’ healthy food choice behavior, based on study results that showcase more healthy eating intentions due to thin-ideal representations for adult, female participants; Stewart and Ogden, 2021) or whether using a thin-ideal influencer only benefits unhealthy food promotion, as it makes these products seem more attractive (Roozen, 2014).

When children and adolescents watch a thin-ideal social media influencer promoting a healthy snack, they might be encouraged to also choose a healthy snack. This might be explained by the mechanism of wishful identification, which refers to the desire to be or act just like the other person (the influencer in this case) (Hoffner, 1996; Schouten et al., 2020). In the context of influencer marketing, tweens look up to the influencers they follow and want to be just (as thin) as those influencers. Hence, they may believe that the influencer attained this ideal body size by consuming healthy foods such as the promoted healthy snack. Thus, through the process of wishful identification (Hoffner, 1996), tweens will also be inclined to choose and consume healthy food to attain the same ideal body type of the influencer.

On the other hand, unhealthy, excessive gain in weight, is based, among other things, on profuse consumption of unhealthy food (e.g., Swinburn et al., 2004; Rosenheck, 2008). And while this is by far not the sole reason for obesity, recent studies showcase that the belief that obesity is connected to unhealthy eating habits is already established in children’s reasoning to explain obesity (Tiggemann and Anesbury, 2000; Gonçalves et al., 2012). Thus, based on these most common conceptions, we assume that if an influencer who is overweight promotes an unhealthy snack, the attractiveness of this unhealthy snack might be diminished and tweens will opt to choose a healthy snack instead. Based on these assumptions, the following hypotheses are formulated:

**H1a**: A thin-ideal influencer promoting a healthy (vs. unhealthy) snack will result in more tweens opting for the healthy snack.
**H1b**: An overweight influencer promoting an unhealthy (vs. healthy) snack will result in more tweens opting for the healthy snack.

### How an Influencer’s Weight Affects Healthy Food Choice Behavior *via* Source Effects

Based on the match-up hypothesis, communicators and their promoted products should fit to be considered persuasive (see e.g., Till and Busler, 2000). Based on existing literature from the field of celebrity endorsement (Bergkvist and Zhou, 2016) and influencer marketing (Breves et al., 2019; De Cicco et al., 2020; Schouten et al., 2020; Naderer et al., 2021), we expect that a seemingly good fit between an influencer and an endorsed product (i.e., product–endorser fit) might positively affect the influencer and the advertised brand. For example, De Cicco et al. (2020) demonstrated with their study that influencer–product congruence increased the influencer’s credibility. In addition, a study by Schouten et al. (2020) showed that a good fit between an influencer and the product embedded in the influencer’s Instagram post resulted in more positive attitudes toward the ad and higher purchase intentions compared to a poor fit between the influencer and the endorsed product. Moreover, the good fit between the influencer and the product also resulted in a higher endorser credibility. Hence, a good product–endorser fit shows to be more favorable for the influencer.

We expect that if the content on an Instagram profile displays thin-ideal images, promotion of a healthy snack is more credible. We base this on study results by Connors et al. (2021) that showcase that for undergraduate students advertisements that feature thin models and healthy (vs. unhealthy) products lead to a greater product–model fit. We, therefore, assume that the promotion of a healthy snack will make the thin-ideal influencer more credible and consequently more effective to tweens compared to promotion of an unhealthy snack by the thin-ideal influencer. In a similar vein, we expect that the thin-ideal social media influencer will not only be perceived as more credible, due to a good product–endorser fit, but that the influencer will also be admired more because the children and adolescents want to be just like the influencer.

Moreover, social media offer the opportunity for consumers to interact with social media influencers. Compared to traditional mass media that only allow for one-way communication, social media allow for two-way communication between social media influencers and their followers. Lou (2021, p. 1) defines the relationship between social media influencers and their followers as *trans*-parasocial relations, which she defines as “a collectively reciprocal, (a)synchronously interactive, and co-created relation between influencers and their captive followers.” In line with the reasoning concerning influencer credibility and influencer admiration, we expect that when there is a good fit between the influencer and the endorsed product (when the thin-ideal influencer promotes a healthy snack), tweens will be more likely to have *trans*-parasocial interactions with this social media influencer as they reward authenticity.

**H2**: Influencer credibility (a), influencer admiration (b), and *trans*-parasocial interaction with the influencer (c) will be higher after exposure to the thin-ideal influencer endorsing a healthy (vs. unhealthy) snack.

However, as for the overweight influencer, we do not expect that a good product-endorser fit will result in more positive source effects. This is because the promotion of an unhealthy product by an overweight influencer might indicate undesired outcomes (i.e., unhealthy weight gain) obtained through consuming unhealthy foods (e.g., Swinburn et al., 2004; Rosenheck, 2008). This expectation is based on the social cognitive theory of Bandura (2001), which explains the improbability that a modeled behavior will be adopted when unfavorable consequences of that behavior are portrayed. Hence, we expect that the promotion of an unhealthy (vs. healthy) snack by an overweight influencer will result in more negative source effects.

**H3**: Influencer credibility (a), influencer admiration (b), and *trans*-parasocial interaction with the influencer (c) will be lower after exposure to the overweight influencer endorsing an unhealthy (vs. healthy) snack.

## Materials and Methods

### Design and Procedure

A two-by-two, between-subjects experimental study (influencer weight: thin-ideal vs. overweight; snack: unhealthy vs. healthy) was conducted. The participants were first exposed to an Instagram profile of an influencer (that either had a thin-ideal weight or was overweight). Then, the tweens saw an individual Instagram post of that same influencer promoting a snack (either unhealthy or healthy). Afterward, the tweens answered the same questionnaire. The experiment was conducted on computers in a classroom setting. The tweens were randomly allocated to one of the four conditions. A researcher was always present to guide the experiment.

### Stimuli Material

To manipulate influencer weight, we created two Instagram profiles and two corresponding Instagram posts of a fictitious influencer. This was the same fictitious influencer in both cases; however, the influencer had a thin-ideal weight in the first Instagram profile and corresponding post, while the influencer was portrayed as overweight in the second profile and corresponding post. Existing pictures of the fictitious influencer were used for the thin-ideal weight conditions, and these pictures were Photoshopped for the overweight influencer conditions. A fictitious influencer was used to avoid confounds with regard to previous experience with, existing attitudes toward and familiarity with the influencer. We made sure that the tweens perceived the fictitious influencer to be an influencer by explaining to them what an influencer is before exposure to the stimulus material [“An influencer is a person with a lot of followers on social media (such as Instagram) who has an influence on others. An influencer uses videos, images, or blogs to promote a product or service”] and by informing them that they would see the Instagram profile and post of Emma Duvillier, who is an influencer. Fictitious influencers are frequently used in experimental research on influencer marketing (e.g., De Veirman et al., 2017; Hudders and De Jans, 2021). In addition, to manipulate the portrayed snack, the influencer was either shown in the individual post holding carrots (i.e., healthy snack) or cookies (i.e., unhealthy snack). These food products were chosen as tweens perceive vegetables (such as carrots) as healthy food, while biscuits or cookies are perceived as snack foods, and thus unhealthy foods (McKinley et al., 2005).

### Participants

In total, 146 tweens between 11 and 13 years (*M*_age_ = 11.33, *SD* = 0.49) participated in the study, and exactly 50% of them were girls. Tweens are young people between 10 and 13 years, and hence the age group between children and adolescents. They are an important target group for this study, as compared to children and adolescents, they are notable for their developmental changes (McArthur et al., 2021). In specific, young people develop healthy lifestyle habits in the tween period and rely on peer relations for their developmental health (Prinstein and Giletta, 2016; McArthur et al., 2021). We randomly selected the tweens from three different high schools in Belgium, a West European country. Institutional ethical approval was obtained and active parental consent was requested for each tween participating in the study. Before the study, it was explained to the tweens that participation was completely voluntary and that they could opt-out at any moment during the study.

### Measures

First, we used ten five-point semantic differentials to measure influencer credibility (Ohanian, 1990; α = 0.86, *M* = 3.44, *SD* = 0.66): “What do you think of [influencer]?” (“unattractive/attractive,” “not classy/classy,” “ugly/beautiful,” “unreliable/reliable,” “lies/tells the truth,” “dishonest/honest,” “untrustworthy/trustworthy,” “not an expert/an expert,” “inexperienced/experienced,” and “unknowledgeable/knowledgeable”).

In addition, we gauged influencer admiration using four items following De Jans et al. (2020; α = 0.84, *M* = 2.38, *SD* = 0.86), using a five-point Likert-scale ranging from “totally disagree” to “totally agree”: “I admire [influencer],” “I look up to [influencer],” “I would like to be just like [influencer],” and “I feel that [influencer] gives direction to my life.”

*Trans*-parasocial interaction (Lou, 2021) was measured using eight items, adapted from the parasocial interaction scale of Lee and Watkins (2016) (α = 0.84, *M* = 2.36, *SD* = 0.78): “I would like to meet [influencer] in person,” “I look forward to watching [influencer]’s pictures,” “If I came across a picture of [influencer], I would definitely watch it,” “When I watch [influencer], it feels like (s)he is my friend,” “I think [influencer] is like a good friend,” “If there were a story about [influencer] in a newspaper or on the Internet, I would read it,” “[influencer] makes me feel comfortable, as if I am with friends,” and “When [influencer] shows me how she feels about a brand, it helps me make up my own mind about the brand.” These items were measured on a five-point Likert-scale ranging from “totally disagree” to “totally agree.”

Finally, we measured food choice behavior using a binary behavioral measure by letting the children choose between a carrot (i.e., healthy snack) and a cookie (i.e., unhealthy snack) (Ngqangashe et al., 2018). More specifically, the tweens were told that they could choose a snack as a thank you for participating in the study and received the chosen snack.

### Data Analysis

To answer H1a and H1b, Chi-square tests were conducted. In addition, two-way analyses of variance (ANOVA) were done to answer H2 and H3.

## Results

### Randomization

The experimental groups did not differ with respect to Instagram involvement [*F*_(3, 145)_ = 1.86, *p* = 0.140].

### Manipulation Checks

The manipulation of the weight of the influencer was measured with the question “What do you think about the weight of the influencer?” (“thin-ideal” or “overweight”). A 95.6% of the tweens that saw the influencer with the thin-ideal weight perceived the influencer as having a thin-ideal weight, which significantly differs from the 4.4% of the children that perceived the influencer as overweight (*z* = 10.6, *p* < 0.001). In addition, 62.8% of the children that saw the overweight influencer perceived the influencer as being overweight, while 37.2% perceived the influencer as having a thin-ideal weight (*z* = 3.2, *p* = 0.001). Hence, the manipulation of influencer weight was on average successful. We also conducted all the analyses with only the participants that correctly answered this manipulation check, but found the same results as reported below.

Furthermore, the product that we displayed in the healthy post-condition (i.e., carrots) was perceived as more healthy (“How healthy do you think the carrots/cookies are?,” measured on a five-point Likert-scale ranging from 1 “not healthy at all” to 5 “very healthy”; *M* = 3.83, *SD* = 0.86) than the product in the unhealthy post-condition [i.e., cookies; *M* = 2.35, *SD* = 0.91; *t*(144) = 10.12, *p* < 0.001]. The manipulation of snack type was also successful.

### Interaction Effect of Snack Type and Influencer Weight on Food Choice Behavior

When tweens saw the influencer with a thin-ideal weight, there was no significant difference (χ^2^ = 0.04, *p* = 0.547) in their choice for the healthy snack after seeing the post with the healthy product (18.2%) vs. after seeing the post with the unhealthy product (20%; *z* = 0.20, *p* = 0.850). H1a cannot be confirmed. However, when the tweens had been exposed to the overweight influencer (χ^2^ = 7.10, *p* = 0.007), they chose significantly more for the healthy snack after seeing the post with the unhealthy product (50%) vs. after seeing the post with the healthy product (21.1%; *z* = 2.70, *p* = 0.008). This confirms H1b.

### Interaction Effects of Snack Type and Influencer Weight on Source Effects

There are no interaction effects of snack type and influencer weight on influencer credibility [*F*_(1, 145)_ = 0.82, *p* = 0.367], influencer admiration [*F*_(1, 145)_ = 0.68, *p* = 0.410], nor on *trans*-parasocial interaction with the influencer [*F*_(1, 145)_ = 2.31, *p* = 0.131]. Hence, H2 and H3 cannot be confirmed. However, we did find main effects of influencer weight on the source effects.

In particular, we found a main effect of influencer weight on influencer credibility [*F*_(1, 45)_ = 10.10, *p* = 0.002]. The participants perceived the influencer as less credible when she was overweight (*M* = 3.28, *SD* = 0.64) compared to when she had a thin-ideal weight (*M* = 3.62, *SD* = 0.64). There was also a main effect of influencer weight on influencer admiration [*F*_(1, 145)_ = 4.38, *p* = 0.038]. In specific, the tweens admired the influencer less when she was overweight (*M* = 2.24, *SD* = 0.77) vs. when she had a thin-ideal weight (*M* = 2.54, *SD* = 0.93). Yet, there was no main effect of influencer weight on *trans*-parasocial interaction with the influencer [*F*_(1, 145)_ = 0.78, *p* = 0.380]: the tweens had the same level of connection with the influencer whether the influencer was overweight (*M* = 2.31, *SD* = 0.72) or whether she had a thin-ideal weight (*M* = 2.42, *SD* = 0.84).

Additional and exploratory analyses (using PROCESS macro, Hayes, 2019, model 4) showed that the source effects did not further affect tweens’ snack choice. In specific, influencer credibility (*B* = 0.44, *SE* = 0.41, *z* = 1.07, *p* = 0.285), influencer admiration (*B* = −0.31, *SE* = 0.39, *z* = −0.79, *p* = 0.430), and *trans*-parasocial interaction with the influencer (*B* = 0.09, *SE* = 0.44, *z* = 0.20, *p* = 0.844) did not affect children’s snack choice.

### Main Effect of Snack Type on Food Choice Behavior

An exploratory analysis was conducted to examine the main effect of snack type on food choice behavior. Of the tweens that we exposed to the healthy snack, 80.3% chose the unhealthy snack and 19.7% chose the healthy snack (*z* = 7.2, *p* < 0.001). Of the tweens that we exposed to the unhealthy snack, 64% chose the unhealthy snack and 36% the healthy snack (*z* = 3.4, *p* < 0.001). Hence, more children chose the unhealthy snack, independent of whether they were exposed to the promotion of the healthy or unhealthy snack. When we compare the percentages of tweens that chose the healthy snack, we can conclude that more tweens selected the healthy snack after exposure to the unhealthy snack (36%) compared to after exposure to the healthy snack (19.7%, *z* = 2.2, *p* = 0.029). Thus, more tweens chose the healthy snack after seeing the unhealthy snack.

## Discussion and Conclusion

The current study investigated whether the weight of a social media influencer could impact tweens’ healthy food choice behavior since previous research on influencer marketing showed that the mere exposure to influencer marketing for healthy foods did not affect children’s and adolescents’ health food choice behavior. Therefore, this study examined how an influencer’s weight as a personal characteristic (thin-ideal vs. overweight) and the promoted snack-type (unhealthy vs. healthy snack) affected tweens’ choice for a healthy snack.

First and in general, the tweens chose more for the unhealthy snack, independent of whether they were exposed to unhealthy vs. healthy food placements in Instagram posts. This is in line with previous research among children and adolescents (Folkvord et al., 2013; Naderer et al., 2018) and can be explained by the inherent human preference for sweets and snacks (Desor et al., 1973; Harris et al., 1990). The results of the study also showed that tweens’ choice for the healthy snack was highest when an overweight influencer promoted an unhealthy snack in an Instagram post (compared to a healthy snack). This indicates that children and adolescents might no longer prefer unhealthy food when they are confronted with the potential assumed negative consequences of consuming it (Tiggemann and Anesbury, 2000; Gonçalves et al., 2012), and therefore, show a contrasting behavior (i.e., choosing the healthy snack). This result is in line with the study of De Jans et al. (2021), which showed that children chose more of a healthy snack when an unhealthy snack was promoted by a social media influencer showing a sedentary lifestyle compared to an athletic lifestyle. Hence, De Jans et al.’s (2021) study examined another possible negative consequence (i.e., sedentary lifestyle) associated with an unhealthy diet. These results may be explained by social cognitive theory, suggesting that when consequences of a particular behavior are considered negative, people will be less likely to adopt the modeled behavior (Bandura, 2001). In both studies, participants even showed contrasting behavior by opting for the opposite food option (i.e., the healthy option).

When the tweens were exposed to the thin-ideal influencer, there was no significant difference in their choice for the healthy snack after seeing the post with the healthy vs. an unhealthy snack. Thus, a thin-ideal influencer did not make a healthy option more attractive to tweens but instead served as an appealing endorser for unhealthy snacks, which is somewhat worrisome. This is not in line with the study of Stewart and Ogden (2021) who reported that using thin-ideal body sizes in media messaging increased women’s intentions toward healthy eating. We could therefore assume that children are less motivated than adults to adopt or copy healthy behaviors due to a confrontation with ideal body images in media messages. We do have to mention that the study of Stewart and Ogden (2021) measured behavioral intentions whereas our study measured actual snack choice behavior by letting the tweens choose between different food options. In sum, while prior research chiefly examined negative health outcomes due to media messages portraying thin-ideal body sizes such as body dissatisfaction and body image disturbance (e.g., Hargreaves and Tiggemann, 2003; Grabe et al., 2008), the current study examined whether thin-ideals in media messages could also result in positive health outcomes among tweens (i.e., healthy food choice behavior). The results of this study show that displaying thin-ideals in influencers’ posts on Instagram does not increase positive health outcomes among tweens.

Finally, the influencer was perceived as less credible and was admired less when she was overweight vs. when she had a thin-ideal weight. This indicates prejudice against certain body types and manifests the predominant “thin equals healthy” stereotype (Connors et al., 2021) that should be addressed in future studies. This again is a somewhat critical result that points to deep-rooted prejudices and misconceptions against overweight and obese people. Overweight and obese people’s weight is still primarily perceived and explained by their lack of self-control when it comes to nutrition (Tiggemann and Anesbury, 2000). This is rather short-sighted and does not appraise the individual circumstances. Showcasing more body diversity in media messages and positively addressing this topic is needed to dismantle these prejudices. These results are also in line with prior research indicating that a social media influencer’s characteristics may affect children’s evaluation of that influencer, whereby personal portrayals of an influencer suggesting an unhealthy diet or lifestyle may negatively affect the influencer’s evaluation. In particular, the study of De Jans et al. (2021) showed that a social media influencer was less admired by children when portraying a sedentary lifestyle compared to an athletic lifestyle in his or her Instagram posts.

### Limitations and Suggestions for Future Research

This study proposes several suggestions for future research. First of all, the current study used a fictitious influencer for the Instagram profiles and individual posts to exclude any biases based on influencer familiarity or likeability, as is done in much extant research on influencer marketing (e.g., De Jans et al., 2021). We expected that tweens would be willing to adopt a portrayed behavior by an influencer due to wishful identification (i.e., the desire to be or act like the influencer, Hoffner, 1996; Schouten et al., 2020); however, we assume that this wishful identification and consequently tweens’ willingness to adopt a modeled behavior of an influencer would be higher when tweens know and follow the influencer. This idea is indeed confirmed by the study of Folkvord et al. (2021), which showed that adults’ healthy food choice behavior was higher when they were exposed to a popular compared to a fictitious influencer *via* parasocial interaction. Thus, we acknowledge that using a fictitious influencer limits the validity of our study. Future research on influencer marketing promoting a healthy diet and lifestyle among children, tweens, and teens should use real and popular influencers. In addition, at the end of the experiment, the tweens were told that they could choose between two snacks as a thank you to participate in the study. These were the two food options that were used in the stimulus materials (cookies and carrots). Hence, it could be that the tweens recognized the study’s purpose and adapted their snack choice accordingly. Therefore, when measuring food choice behavior or snack choice, future research could let the participants choose between different healthy and unhealthy options. In this way, we also reduce the possibility that the tweens choose a certain snack solely because of their liking or disliking of certain food products.

### Implications

This study shows that exposure to a thin-ideal influencer did not affect tweens’ choice for healthy vs. unhealthy foods. Hence, we suggest that using thin-ideal social media influencers does not stimulate a healthy diet among tweens. However, exposure to an overweight influencer promoting unhealthy snacks can positively affect children’s choice of healthy food. These results could be explained by contrast effects, as the overweight influencer is also perceived as less credible and is admired less by the tweens. Based on this main result, it is difficult to draw a concrete recommendation for marketers or public policies when it comes to promoting healthy food to children and adolescents, as our results would suggest that the best way to promote a healthy diet is by using an overweight influencer promoting an unhealthy food product. Thus, we believe that it is not advisable to promote healthy food to children through the endorsement of unhealthy food by an overweight influencer, as this may perpetuate the stereotypes regarding overweight people in that people who do not have a thin ideal are unhealthy and eat unhealthy food (Connors et al., 2021). We do suggest influencers to be cautious with promoting food products in general, as promoting unhealthy food can have an impact on their followers’ unhealthy food behavior and lifestyle. Recent content analyses, however, show that influencers often abundantly endorse unhealthy food products in their videos (Coates et al., 2019c; Martínez-Pastor et al., 2021). Therefore, influencers should be made aware of the ethical responsibility they have when choosing to promote products. In addition, it would be helpful to strengthen initiatives that regulate the advertising of unhealthy foods to children. In Europe, the European Pledge initiative already takes measures to limit the endorsement of unhealthy foods to children under twelve. More specifically, this agreement prohibits the endorsement of foods that do not meet the predefined nutritional criteria on influencer accounts targeting young children. Some argue, however, that the pledge cannot sufficiently protect children from exposure to unhealthy foods (Neyens and Smits, 2017). Social media are still a relatively unregulated space in this regard, and marketers should be held accountable when targeting children with unhealthy products, especially since the negative consequences of marketing unhealthy foods to children are well known (Folkvord et al., 2022). Given the volatile nature of social media content, it is difficult to monitor and penalize breaches. Future research is necessary to examine how social media influencers can be deployed to promote a healthy diet. For example, it could be investigated whether increasing children’s and adolescents’ food literacy through influencers’ social media posts can enhance children’s and adolescents’ healthy food behavior. In addition, we suggest that an influencer’s evaluation does not depend on which food product they promote considering their body type, as we did not find interaction effects of influencer weight and snack type on the influencer effects. Nonetheless, the results indicate the need to tackle deep-rooted prejudice and assumptions about overweight and obese people in media messages, as our study shows that an influencer was evaluated less positively when being overweight. For example, influencers with different body types could be deployed for the promotion of exercise campaigns among children by the government, or brands for healthy products or services could hire influencers with different body types to promote their products on the influencers’ social media channels.

## Data Availability Statement

The raw data supporting the conclusions of this article will be made available by the authors, upon request.

## Ethics Statement

The studies involving human participants were reviewed and approved by the Ethical Commission of the Faculty of Political and Social Sciences, University of Ghent. Written informed consent to participate in this study was provided by the participants’ legal guardian/next of kin.

## Author Contributions

SDJ contributed to the conceptualization of the study, conducted the formal analyses, wrote the original draft of the manuscript, and contributed to manuscript revision. LH contributed to the conceptualization of the study, the formal analyses, the writing of the manuscript, and manuscript revision. BN contributed to the writing of the manuscript and manuscript revision. VDP contributed to the conceptualization of the study and collected the data. All authors contributed to and approved the submitted article.

## Conflict of Interest

The authors declare that the research was conducted in the absence of any commercial or financial relationships that could be construed as a potential conflict of interest.

## Publisher’s Note

All claims expressed in this article are solely those of the authors and do not necessarily represent those of their affiliated organizations, or those of the publisher, the editors and the reviewers. Any product that may be evaluated in this article, or claim that may be made by its manufacturer, is not guaranteed or endorsed by the publisher.
